# Supplementary material for: Complexome profiling of the Chlamydomonas psb28 mutant reveals TEF5 as an early PSII assembly factor
Source: Plant Cell. 2025 Mar 17;37(6):koaf055. doi: 10.1093/plcell/koaf055 (PMC12164590; doi:10.1093/plcell/koaf055)
Supplement: koaf055_Supplementary_Data [file koaf055_supplementary_data.zip › psb28_interactive.zip!/plots/intensity/qualityPlots/peptides/SHMT1_Cre16.g664550.t1.2.html]

### Cre16.g664550.t1.2

Trivial name: SHMT1  
SeqCoverage [median]: 60.00  
Mass: 57.17 kDa  
  
 

| Sequence | MissedCleavage | Mass [Da] | wtIntensity | muIntensity | Score |
| --- | --- | --- | --- | --- | --- |
| AHVTWPK | 0 | 837.45 | 0.00 | 427.05 | 77.75 |
| AVNIAVDLK | 0 | 941.55 | 5029.48 | 13199.19 | 92.44 |
| AVNIAVDLKK | 1 | 1069.65 | 3007.58 | 7889.89 | 89.83 |
| DVETFAMR | 0 | 967.44 | 3955.82 | 7176.60 | 127.12 |
| DVETFAMRFPTIGFDK | 1 | 1872.91 | 3735.09 | 0.00 | 61.44 |
| ESTPDINALKK | 1 | 1214.65 | 25739.05 | 26833.87 | 106.35 |
| FPTIGFDK | 0 | 923.48 | 1958.80 | 9752.39 | 89.55 |
| FSQDASR | 0 | 809.37 | 16268.50 | 19370.51 | 161.60 |
| GAMIFYR | 0 | 856.43 | 886.92 | 0.00 | 81.00 |
| GFVEKDFEQVAEFVDR | 1 | 1913.92 | 949.34 | 0.00 | 106.38 |
| GLELIPSENFVSASVMEAVGSVMTNK | 0 | 2708.34 | 841.61 | 0.00 | 47.38 |
| HYDYAR | 0 | 823.36 | 1149.70 | 10852.37 | 148.40 |
| IMALDLPHGGHLSHGYQTDTK | 0 | 2290.12 | 0.00 | 1144.72 | 53.78 |
| ISATSIYFEQMPYR | 0 | 1704.82 | 6281.54 | 4667.91 | 129.89 |
| KISATSIYFEQMPYR | 1 | 1832.91 | 821.70 | 0.00 | 55.19 |
| LIVAGASAYTR | 0 | 1120.62 | 28147.88 | 45846.23 | 150.35 |
| LNEETGLIDYDMLEK | 0 | 1781.84 | 11350.48 | 20264.25 | 213.99 |
| LVSGGTDNHIVLVDLRPK | 1 | 1932.08 | 15530.22 | 40181.29 | 132.47 |
| MGSPALTSR | 0 | 918.46 | 23392.58 | 23627.56 | 131.22 |
| NTVPGDVSALVPGGLR | 0 | 1550.84 | 8500.81 | 20473.90 | 128.85 |
| QAATPEFK | 0 | 890.45 | 1626.46 | 682.13 | 84.92 |
| SYQQQVLSNSQALAGALAK | 0 | 1976.03 | 11851.43 | 25304.64 | 173.87 |
| TAVLFRPK | 1 | 930.57 | 1761.94 | 0.00 | 116.54 |
| TGKPINYDIEDK | 1 | 1391.69 | 0.00 | 9462.28 | 96.87 |
| VLELAHIAANK | 0 | 1177.68 | 13035.39 | 21474.93 | 93.10 |
| VLELAHIAANKNTVPGDVSALVPGGLR | 1 | 2710.51 | 1415.44 | 0.00 | 65.75 |
| VLNAGLAEVDPDLFDIIEKEK | 1 | 2327.23 | 2048.39 | 0.00 | 116.39 |
| YSEGYPGAR | 0 | 998.45 | 8431.14 | 10419.52 | 144.38 |
| YYGGNEFIDQAER | 0 | 1560.68 | 876.27 | 2567.62 | 108.70 |
|  |  |  | 198593.6 | 321618.8 |  |
